# Association of Inherited Copy Number Variation in *ADAM3A* and *ADAM5* Pseudogenes with Oropharynx Cancer Risk and Outcome

**DOI:** 10.3390/genes13122408

**Published:** 2022-12-19

**Authors:** Juliana Carron, Caroline Torricelli, Janet Keller Silva, Yichuan Liu, Renata Pellegrino, Carmen Silvia Passos Lima, Gustavo Jacob Lourenço

**Affiliations:** 1 Laboratory of Cancer Genetics, School of Medical Sciences, University of Campinas, Campinas, São Paulo 13083-888, Brazil; 2 Center for Applied Genomics, The Children’s Hospital of Philadelphia, Philadelphia, PA 19104, USA; 3 Department of Pediatrics, The Children’s Hospital of Philadelphia, University of Pennsylvania, Philadelphia, PA 19104, USA; 4 Department of Anesthesiology, Oncology, and Radiology, School of Medical Sciences, University of Campinas, Campinas, São Paulo 13083-887, Brazil

**Keywords:** oropharynx squamous cell carcinoma, copy number variation, pseudogene, *ADAM3A*, *ADAM5*, microRNA

## Abstract

Inherited copy number variations (CNVs) can provide valuable information for cancer susceptibility and prognosis. However, their association with oropharynx squamous cell carcinoma (OPSCC) is still poorly studied. Using microarrays analysis, we identified three inherited CNVs associated with OPSCC risk, of which one was validated in 152 OPSCC patients and 155 controls and related to pseudogene-microRNA-mRNA interaction. Individuals with three or more copies of *ADAM3A* and *ADAM5* pseudogenes (8p11.22 chromosome region) were under 6.49-fold increased risk of OPSCC. *ADAM5* shared a highly homologous sequence with the *ADAM9* 3′-UTR, predicted to be a binding site for miR-122b-5p. Individuals carrying more than three copies of *ADAM3A* and *ADAM5* presented higher *ADAM9* expression levels. Moreover, patients with total deletion or one copy of pseudogenes and with higher expression of miR-122b-5p presented worse prognoses. Our data suggest, for the first time, that *ADAM3A* and *ADAM5* pseudogene-inherited CNV could modulate OPSCC occurrence and prognosis, possibly through the interaction of *ADAM5* pseudogene transcript, miR-122b-5p, and *ADAM9*.

## 1. Introduction

Oropharyngeal (OP) squamous cell carcinoma (SCC) is a subtype of head and neck (HN) SCC, and its main risk factors are tobacco and alcohol consumption [1]. Human papillomavirus (HPV) infection is also a consistent risk factor, and it seems to be a favorable prognostic factor for these patients [2]. Most OPSCC are diagnosed as an advanced disease [3,4,5], and cisplatin (CDDP), whether associated or not with radiotherapy (RT), has been used in HNSCC patients’ treatment [6].

Copy number variations (CNVs) are defined as the loss or gain of a DNA segment of one kilobase (kb) or larger that is present at a variable copy number in comparison with a reference genome assembly [7]. CNVs have been broadly documented in tumor (somatic CNVs) [8] and germline (inherited CNVs) cells, and they represent a critical factor driving OPSCC development and outcome [9,10].

Somatic CNVs have been more studied in general HNSCC than inherited ones. For instance, somatic cells of OPSCC featured focal amplification of the 3q26-28 region, containing the transcription factor genes *TP63* and *SOX2*, and *PIK3CA* oncogene [11,12]; the 11q13 region, containing the cell cycle regulator gene *CCND1*, the tumor suppressor gene *FADD*, and the *CTTN* oncogene [11,13]; the 11q22 region, containing the *BIRC2* and *YAP1* oncogenes; and the 20q11.22 region, containing the cell cycle regulator gene *E2F1* [11]. Somatic cells of OPSCC also featured loss of the 14q32.31 region, containing the *TRAF3* gene, involved in antiviral immune response [11]; and loss of the 4q35.2, 5q35.3, 9p21.3, 9q34.3, 17p13.1, and 18q21.2 regions, containing the *FAT1*, *NSD1*, *CDKN2A*, *NOTCH1*, *TP53*, and *SMAD4* tumor suppressor genes, respectively [11,14,15]. Moreover, loss of the 5q35.1 region, containing the *FGF18* oncogene, led to worse overall survival (OS) of OPSCC patients; but loss of the 16q24.3 region, containing tumor suppressor *CDK10*, and the 3p25.3 region, containing the *RAD18* gene involved in telomere maintenance, led to better OS for these patients [9].

Inherited CNVs can provide valuable information for cancer risk prediction and prognosis evaluation, and they have been identified in several cancers [8,16,17]. In HNSCC, the nasopharyngeal SCC is the only subtype which has been studied so far, whereas the amplification of the 1q32 region containing the *MAPKAPK2* oncogene [18]; the 11q14.3 region, containing the glutamatergic signaling *GRM5* [19]; and the deletion of the 6p21.3 region, containing the *MICA* and *HCP5* immune genes [20] were associated with nasopharyngeal SCC susceptibility. It is interesting to note that several genes affected by inherited CNVs have already been implicated in carcinogenesis themselves, indicating that these CNVs are related to cancer predisposition [8].

For a long time, pseudogenes were considered non-functional (“junk”) regions of the DNA. However, thanks to advancements in genomics research, they are now recognized to regulate gene expression [21]. Pseudogene-derived transcripts, containing microRNA(miRNA)-binding elements, can exert the regulatory control of ancestral or other genes’ expression levels by competing for miRNAs, acting as critical modulators in cancer development and progression [21,22].

Particularly in HNSCC, loss of copy number of the pseudogene *PTENP1* (9p13.3 region) inhibited cell proliferation and tumor cell migration, possibly by acting as an miRNA competitor [23]. The increased expression of the pseudogene-derived long non-coding(lnc)-RNA *FTH1P* facilitated oral SCC progression by acting as a molecular sponge of miR-224-5p in order to modulate *FZD5* oncogene expression [24] and by enhancing PI3K/Akt/GSK3b/Wnt/β-catenin signaling, which are involved in tumor cell migration and invasion [25]. The increased expression of *FTH1P* also promoted cell proliferation, migration, and invasion in laryngeal SCC tissues [26]. Moreover, the amplification of the 8p11.23–22 region, which contains the pseudogene *ADAM5*, was described in oral tumor samples [27]. The *ADAM* gene family encodes the proteins enrolled in cell adhesion, migration, and proliferation, and has been associated with carcinogenesis [28].

Since inherited CNVs in genes with importance in OPSCC are still poorly identified, we conducted a two-stage association study on patients with OPSCC and healthy controls from the southeastern region of Brazil and identified an inherited CNV in *ADAM3A* and *ADAM5* pseudogenes-associated with risk of OPSCC and prognosis.

## 2. Materials and Methods

### 2.1. Study Population

This study was conducted in two steps. Duriong step 1 (pilot-study), 49 patients and 49 controls were analyzed with the purpose of identifying CNVs with importance in OPSCC development, and in step 2, three CNVs were selected for data validation in 152 OPSCC patients and 155 controls.

More details about the dataset of the cohort of OPSCC patients and the controls can be found in Carron et al. [5]. The study was approved in keeping with the institutional review board guidelines (numbers: 424/2016 and 1,438,601) and according to the Declaration of Helsinki. All patients and controls provided written informed consent to participate in this study.

For survival analysis, we selected 139 patients; 13 out of 152 patients were sent to other services for treatment and follow-up, and no consistent clinical information could be retrieved. Patients were treated according to the institutional protocol, based on conventional procedures for CDDP, RT, and surgery [5]. The follow-up of patients was performed at 3-month intervals. The end of the follow-up period was November 2022. Ninety-one patients (67.0%) were submitted to chemotherapy (CT) and RT-combined treatment; 30 patients (22.0%) received CT, RT, and were submitted to surgery; 6 patients (4.4%) received RT and surgery; 4 patients (2.9%) received CT only; 2 patients (1.5%) received RT only, and 3 patients (2.2%) were submitted to surgery only. It was not possible to obtain consistent information about 3 patients’ therapeutics enrolled in survival analysis (*n* = 139).

### 2.2. Step 1: Screening of CNVs, Candidate DNA Region Choice, and CNVs Selection

Ninety-eight individuals were studied in the first step. Genomic DNA from the peripheral blood of 49 base-of-tongue (BT) SCC patients and 49 controls was genotyped individually using DNA high-resolution microarrays containing 420,000 CNVs (Genome-Wide Human SNP Array 5.0, Affymetrix, Santa Clara, CA, USA) as detailed in Carron et al. [5].

The PennCNV software package was used to call CNVs based on the intensity data from the microarrays, including the log R ratio and the B allele frequency for each CNV [29]. The software BEDTools for genomic arithmetic integrated the CNV data from the sample analysis, merging the CNV regions that were common among them [30]. CNV calls were grouped into loci at least one kb in length. In order to select a CNV for further validation, a confidence score of 10 probes minimum was used as a threshold to classify reliable CNV calls [31], and, in order to restrict the analysis, at least 5 patients had to be identified with the CNV (copy number gain or loss). Next, the CNVs were selected by the presence of carcinogenesis genes in the CNV region, the process being performed using the Database for Annotation, Visualization, and Integrated Discovery (david.ncifcrf.gov/ accessed on 16 February 2021)) and the Kyoto Encyclopedia of Genes and Genomes pathway maps (https://www.genome.jp/kegg/ accessed on 16 February 2021).

As a result, we selected the 4q13.2, 8p11.22, and 20p13 regions harboring, respectively, the *UGT2B17* gene, *ADAM3A* and *ADAM5* pseudogenes, and *SIRPB1* gene for further validation.

### 2.3. Step 2: Validation of CNVs in OPSCC Risk

A total of 307 individuals were analyzed in Step 2, including the 98 individuals analyzed preliminary in Step 1. Genomic DNA from leukocytes of the peripheral blood of 152 OPSCC patients and 155 controls was analyzed through real-time polymerase chain reaction (PCR) using TaqMan Copy Number Assays (assay reference: Hs04875988_cn, Hs03274455_cn, and Hs04494662_cn for the 4q13.2, 8p11.22, and 20p13 chromosome regions, respectively; Applied Biosystems, Waltham, MA, USA), following the manufacturer’s instructions. For the internal control, a predesigned TaqMan Copy Number Reference Assay RNase P (assay reference: 4403328; Applied Biosystems, USA) was used. Positive and negative controls were used in all reactions. The qPCR analysis was carried out using the 2^−ΔΔCt^ cycle threshold method [32], and the copy number was calculated using the Copy Caller™ Software v2.0 (Applied Biosystems, USA). A confidence level of 95% and a |z-score| value of less than 1.75 was applied in order to cull the CNVs. Copy number loss was defined as being equal or less than 1 copy per cell and copy number gain as equal or more than 3 copies per cell. Values of 20% of the samples were repeated in separate experiments with 100% agreement. As a result, *ADAM3A* and *ADAM5* CNV were selected for further analysis.

### 2.4. Potential Pseudogene-miRNA-mRNA Interaction

After CNV validation, we searched for a potential pseudogene-miRNA-mRNA interaction in order to test the hypothesis that pseudogene-derived transcripts could compete for miRNA binding, modulating another type of gene expression [21]. First, we searched for any *ADAM* family genes sharing a highly homologous sequence to *ADAM3A* or *ADAM5* pseudogenes, especially in the 3′-untranslated region (3′-UTR), using the Basic Local Alignment Search Tool (blast.ncbi.nlm.nih.gov). As a result, the *ADAM9* gene shared a highly homologous sequence with the *ADAM5* pseudogene. Next, miRWalk 2.0 [33] was employed to identify potential miRNAs binding to the 3′-UTR of the *ADAM9* gene. We selected the miRNAs that match at least seven nucleotides in the seed region (position 2–8 of miRNA). As a result, we selected the miR-122b-5p.

### 2.5. ADAM9 Expression by qPCR

In order to assess the potential pseudogene-miRNA-mRNA interaction, we analyzed *ADAM9* gene expression. The total RNA from leukocytes of the peripheral blood of 26 OPSCC patients with a distinct copy number of the *ADAM3A* and *ADAM5* pseudogenes (6 patients with copy number loss, 15 with two copies, and 5 with copy number gain) was extracted with TRIzol reagent (Life Technologies, Carlsbad, CA, USA), following the manufacturer’s instructions. cDNA was generated using SuperScript III reagents (Life Technologies, USA). Experiments were performed using SYBR Green PCR Master Mix reagents (Applied Biosystems, USA) and specific primers for the *ADAM9* gene (forward: 5′-GAATTCAGGAGCATCGGGTTC-3′, and reverse: 5′-CCCAGCGTCCACCAACTTAT-3′), in triplicate per sample, and a negative control without template was included in each plate. The relative expression level of *ADAM9* was normalized to the reference housekeeping gene actin β level (forward: 5′-AGGCCAACCGCGAGAAG-3′, and reverse: 5′-ACAGCCTGGATAGCAACGTACA-3′) using the 2^−ΔΔCt^ cycle threshold method [32]. Values of 20% of the samples were repeated in separate experiments with 100% agreement. Results were expressed in arbitrary units (AUs).

### 2.6. Statistical Analysis

The association between disease statuses, BTSCC patients vs. controls, and CNVs for Step 1 was performed using the software ParseCNV, which uses a genome-wide segment-based scoring approach [31]. Afterward, Fisher’s exact test was used to compare the CNV frequency among cases and controls.

Receiver-operating characteristic (ROC) curve analysis was used to determine the optimal cut-off score for the *ADAM3A* and *ADAM5* pseudogenes’ copy number alterations (scores) to survival status using the 0, 1 criterion [34]. The sensitivity and specificity were plotted in order to generate the area under the ROC curve (AUC) against the survival status (alive vs. progression, relapse, or death by disease).

The differences between groups were analyzed by χ^2^ or Fisher’s exact test. Multivariate analysis using the logistic regression model served to obtain age- and tobacco status-adjusted crude odds ratios (ORs) with a 95% confidence interval (CI), and to assess the associations between CNVs and OPSCC in step 2.

Either Chi-square or Fisher’s exact test was used to evaluate the possible associations between clinical characteristics, tumor aspects, and the selected CNVs. To evaluate the robustness of our results, the false discovery rate was computed to reflect the expected ratio of false-positive findings to the total number of significant findings.

Considering continuous variables, data sets were probed for normality using Shapiro–Wilk’s test. For *ADAM9* gene expression, the data set assumed normal distribution, and a *t*-test was used for analysis.

For survival analysis, the event-free survival (EFS) was calculated from the date of diagnosis to the date of progression, relapse, death caused by the disease, or last follow-up. OS was calculated from the date of diagnosis until the date of death, resulting in any cause, or last follow-up. EFS and OS times were calculated using Kaplan–Meier estimate probabilities, and the differences between survival curves were analyzed by the log-rank test. The prognostic impacts of clinicopathological aspects and each genotype in the survival of OPSCC patients were examined using Cox proportional hazard ratio (HR) regression. In a second step, all variables with *p* < 0.10 were included in a multivariate Cox regression (backward conditional step-wise selection). In the multivariate Cox regression, *p* values < 0.05 were considered statistically significant.

As a complementary survival analysis, we assessed the Kaplan–Meier Plotter analysis (kmplot.com/analysis/ (accessed on 22 March 2021), an online database established from the gene expression data and survival information of cancer patients from the Gene Expression Omnibus (GEO) database, in order to evaluate the prognostic value of *ADAM3A* and *ADAM5* and miR-122b-5p in general HNSCC patients, since it was not possible to stratify for OPSCC [35]. A Kaplan–Meier survival plot and log-rank *p* were directly determined and displayed by the database.

For all statistical tests, the significance is two-sided and achieved when *p* values were < 0.05. All tests were conducted using the SPSS 21.0 software (SPSS Incorporation, Chicago, IL, USA).

## 3. Results

### 3.1. Study Population

The sociodemographic data of 152 OPSCC and 155 controls are presented in Supplementary Table S1. The control individuals were younger than OPSCC patients (median age: 46 vs. 56 years, *p* < 0.001), and the number of tobacco and alcohol users was higher among patients than in controls (98.0% vs. 26.5%, *p* < 0.001; 92.8% vs. 63.9%, *p* < 0.001; respectively). The differences in the age and pattern of tobacco and alcohol use of individuals from each group were corrected in all comparisons of genotype frequencies by pertinent statistical analyses.

The frequencies of tumor characteristics of OPSCC patients are shown in Supplementary Table S2. HPV 16 was positive in only 1 out of 42 OPSCC patients analyzed in the study. Most patients presented tumor stage IV (67.1%), moderately differentiated tumors (78.3%), and tumors located in the BT (47.4%).

### 3.2. Step 1: Analysis of Screening and Selection of CNVs

After screening of CNVs (49 BTSCC patients vs. 49 controls), we observed 1666 copy number gain and 949 copy number loss having at least 1 kb in length associated with BTSCC risk (Supplementary Dataset), but only eight CNVs were identified in at least five patients (Supplementary Table S3). Among them, the *GSTT1* gene, enrolled in carcinogen detoxification, is well known to play a role in HNSCC, so it was excluded from the validation selection [36]. Next, we selected three CNVs that harbor genes related to carcinogenesis pathways for validation. These CNVs regions included: the *UGT2B17* gene (4q13.2 chromosome, position: 69,085,497–69,116,840; range: 31.343 bp); the *ADAM3A* and *ADAM5* pseudogenes (8p11.22 chromosome, position: 39,291,338–39,517,385; range: 226.047 bp); and the *SIRPB1* gene (20p13 chromosome, position: 1,525,985–1,548,689; range: 22.704 bp). Data on the genome association were deposited at the GEO database (www.ncbi.nlm.nih.gov/geo/ (accessed on 20 October 2020)) with accession number GSE46812.

CNVs were dichotomized as total deletion, 1 or 2 copies vs. 3 or more copies to compare scores of CNVs, case and control, and clinical and pathological data. Analyzing the ROC curve, the cut-off value one for CNV (AUC = 0.335, *p* = 0.001, sensitivity = 64.9%, and specificity = 85.5%) was also included in the analyses.

### 3.3. Step 2: Analysis of Validation of CNVs

Three or more copies of the *ADAM3A* and *ADAM5* pseudogenes were detected more frequently in OPSCC patients than in controls (16.4% vs. 4.5%, *p* = 0.002). Individuals carrying three or more copies of *ADAM3A* and *ADAM5* were under 6.49-fold increased risk of OPSCC than carriers of two copies or fewer (Table 1). No associations between *UGT2B17* and *SIRPB1* CNVs were seen during the validation of these CNVs (Table 1).

Subsequent analyses were performed only for *ADAM3A* and *ADAM5* CNV due to the limited individual carriers of *UGT2B17* and *SIRPB1* with a copy number different than two.

### 3.4. Association between Clinical and Tumor Aspects with CNVs

No associations between *ADAM3A* and *ADAM5* CNVs were seen in terms of clinical or tumor aspects (Table 2).

### 3.5. Potential Pseudogene-miRNA-mRNA Interaction

**Figure 1:**
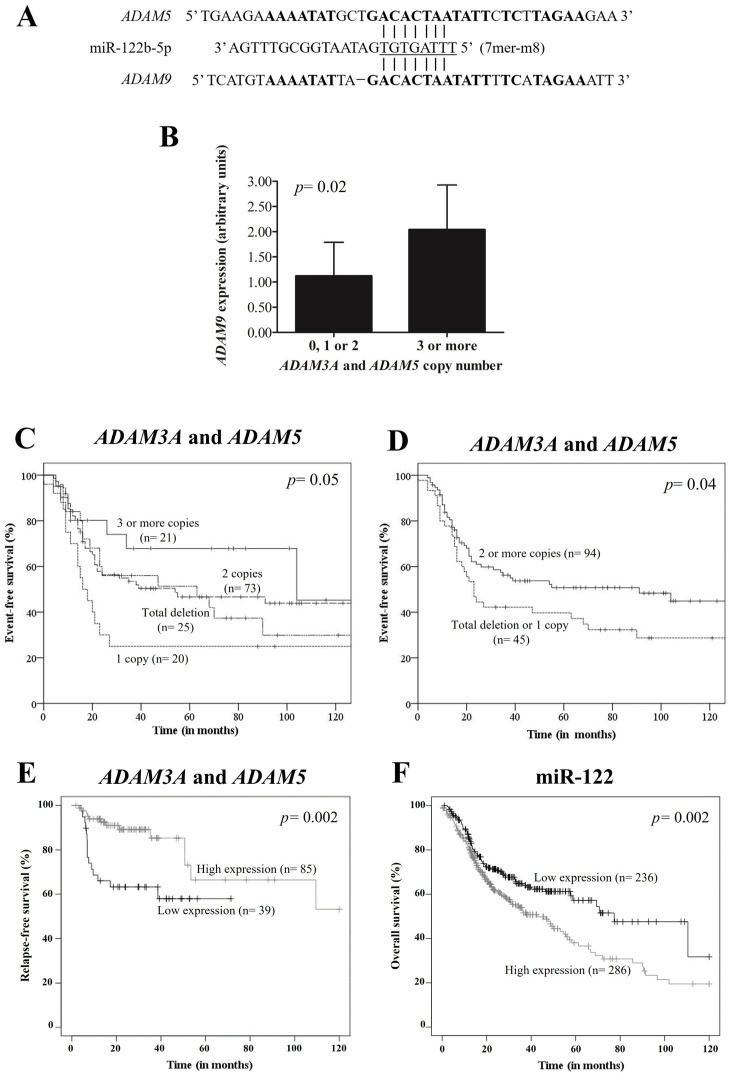
Potential pseudogene-microRNA(miRNA)-mRNA interaction and head-and-neck squamous cell carcinoma (HNSCC) prognosis. (**A**) Predicted miRNA miR-122b-5p binding site at *ADAM5* pseudogene-derived transcript and at *ADAM9* 3′-unstranslated region (7mer-m8 site). The miRNA “seed” region is presented in underlined font. The homologous sequence between *ADAM5* and *ADAM9* is represented in bold letters. (**B**) *ADAM3A* and *ADAM5* pseudogenes copy number variation and *ADAM9* expression in oropharyngeal squamous cell carcinoma (OPSCC) patients. The mean mRNA expression level was higher in OPSCC patients with copy number gain of the *ADAM3A* and *ADAM5* pseudogenes (*n* = 5) (2.04 arbitrary units (AUs) ± standard deviation (SD): 0.89) than carriers of total deletion, one, or two copies (*n* = 21) (1.12 AUs ± SD: 0.67, *p* = 0.02). (**C**,**D**) Probability of event-free survival (EFS) of 139 OPSCC patients stratified by *ADAM3A* and *ADAM5* copy number variation. (**C**) The Kaplan–Meier curve suggests that CNVs status seems to have an association with EFS (*p* = 0.05). (**D**) The Kaplan–Meier curve indicates lower EFS in patients with total deletion or one copy of *ADAM3A* and *ADAM5* pseudogenes (19.1% vs. 50.7%, *p* = 0.04) when compared with carriers of two or more copies. (**E**,**F**) Prognostic value of *ADAM3A* and *ADAM5*, and miR-122 in 124 and 522 HNSCC patients, respectively, according to the Kaplan–Meier Plotter online database. (**E**) Lower expression of pseudogenes *ADAM3A* and *ADAM5* indicated lower relapse-free survival (RFS) in HNSCC patients. (**F**) Although RFS data was not available for miRNAs, higher expression of miR-122b-5p was associated with lower overall survival in HNSCC patients.

We explored the potential regulatory mechanisms of pseudogenes *ADAM3A* and *ADAM5* among which the hypothesis of the pseudogene-derived transcripts could compete with another *ADAM*-family gene for miRNA binding may be a representative approach. Among the ADAMs genes, *ADAM9* and *ADAM33* presented a highly homologous sequence at the 3′-UTR with the *ADAM5* pseudogene (Figure 1A). We selected *ADAM9* for further validation and miRNAs search due to its potential association with *ADAM5*, as previously reported [26]. We identified the miR-122b-5p as a potential miRNA for *ADAM9* and *ADAM5* binding (Figure 1A).

### 3.6. ADAM9 Expression by qPCR

Higher *ADAM9* mRNA expression levels were seen in OPSCC patients with three or more copies of the *ADAM3A* and *ADAM5* pseudogenes (*n* = 5) than carriers with total deletion or one or two copies (*n* = 21) (2.04 AUs ± standard deviation (SD): 0.89 vs. 1.12 AUs ± SD: 0.67, *p* = 0.02) (Figure 1B).

### 3.7. Survival Analysis of OPSCC Patients

The median follow-up of the 139 patients enrolled in survival analysis was 39 months (range 4–200 months). The final status of patients was established in November 2022. At this time, 25 (18.0%) patients were alive without disease, 8 (5.7%) patients were alive with disease, 72 (51.8%) patients had died due to disease, and 34 (24.5%) patients had died of unrelated causes. The 5-year EFS and OS rates were 47.0% and 42.0%, respectively.

When we stratified the patients by *ADAM3A* and *ADAM5* copy number (total deletion or one, or two, or three or more copies), we observed that CNVs status seems to have an association with EFS (*p* = 0.05) (Figure 1C).

At 60 months of follow-up, lower EFS was observed in patients with advanced tumor stage (37.8% vs. 69.2%, *p* = 0.002) when compared with others, and in patients with total deletion or one copy of the *ADAM3A* and *ADAM5* pseudogenes (39.7% vs. 50.7%, *p* = 0.04) when compared with carriers of two or more copies (K–M estimates) (Figure 1D). The significance of the differences between groups remained the same in univariate Cox analysis. After multivariate Cox analysis, advanced tumor stage (HR: 2.34, 95% CI: 1.37–4.00, *p =* 0.002) and total deletion or one copy of the *ADAM3A* and *ADAM5* pseudogenes (HR: 1.68, 95% CI: 1.07–2.66, *p* = 0.02) were found to be predictors of poor EFS (Table 3).

At 60 months of follow-up, lower OS was observed only in patients with advanced tumor stage (32.0% vs. 63.6%, *p* < 0.001) when compared with others (K–M estimates). *ADAM3A* and *ADAM5* copy number loss or gain did not influence the OS of our 139 OPSCC patients (Table 3).

Additionally, the prognostic value of *ADAM3A* and *ADAM5*, and miR-122b-5p in 124 and 522 HNSCC patients, respectively, was further evaluated using the Kaplan–Meier Plotter online database. We found that lower expression of the pseudogenes *ADAM3A* and *ADAM5* (Figure 1E) indicated lower relapse-free survival (RFS) in general HNSCC patients (*p* = 0.002), and higher expression of miR-122b-5p was associated with lower OS in general HNSCC patients (*p* = 0.002) (Figure 1F).

## 4. Discussion

We investigated herein whether inherited CNVs alter the risk and prognosis of OPSCC patients. We initially observed that CNVs in the 4q13.2 region, containing the *UGT2B17* gene; the 8p11.22 region, containing the *ADAM3A* and *ADAM5* pseudogenes; and the 20p13 region, containing the *SIRPB1* gene, were identified in BTSCC patients in a pilot study (Step 1). However, after validation in a large cohort of OPSCC patients (Step 2), the 4q13.2 and 20p13 regions did not confirm their association with OPSCC risk.

In HNSCC, only one study with the *UGT2B17* gene was found [37]. Researchers suggested that *UGT2B17* somatic deletion could modify the effects of smoking on *TP53*-mutations, increasing the relapse rates among HPV-positive HNSCC patients [37]. However, we did not find any relationship between the 4q13.2 region and OPSCC risk. A possible explanation for our distinct result may be the prevalence of HPV-negative patients in our cohort and that Mafune et al. [37] analyzed tumor cells for somatic mutation while in our study, we searched for inherited CNVs in patients’ blood samples.

A complex pattern of amplification and deletion of the 20p13 region was previously associated with oral SCC risk [27]. This is the only study focusing on the potential role of *SIRPB1* in HNSCC, although the researchers did not find an association between *SIRPB1* and oral SCC risk after validation in tumor samples [27], as was the case in our study.

We observed that the gain of more than three copies of the 8p11.22 region, which contains the *ADAM3A* and *ADAM5* pseudogenes, was associated with OPSCC risk. Moreover, Vincent–Chong et al. [27] detected, in tumor samples, amplification in the *ADAM5* region (8p11.23-p11.22) associated with oral SCC risk [27]. In contrast, two studies identified the loss of the somatic copy number of the 8p11 region to be associated with oral SCC risk in a male-prevalent cohort [38] and in oral and laryngeal SCC cell lines [39]. To the best of our knowledge, there are no studies focusing on the role of the referred CNV in the development or progression of pharyngeal SCC, neither somatic nor in an inherited way. In addition to somatic CNVs in tumor cells, identifying inherited cancer susceptibility in an individual may be used clinically to facilitate diagnosis and to recognize high-risk patients that should receive a closer follow-up.

*ADAM3A* and *ADAM5* are classified as pseudogenes once they do not seem to produce a specific protein [40]. The prevalent existence of pseudogenes indicates that they may play a key role in basic physiology and disease progression, including that of cancer [22,41]. The ways in which *ADAM3A* and *ADAM5* may mediate tumorigenesis in OPSCC are still unclear, but pseudogenes can interact with parental genes or other gene loci, altering their sequences and/or transcriptional activities [41].

In order to support the hypothesis that pseudogene-derived transcripts could compete for miRNA binding, we searched for a potential pseudogene-miRNA-mRNA interaction. We found that the *ADAM5* pseudogene shared a highly homologous sequence at 3′-UTR of the *ADAM9* gene, and this sequence was predicted to be a binding site for miR-122b-5p. In fact, we observed that OPSCC patients with more than three copies of the *ADAM3A* and *ADAM5* pseudogenes presented higher *ADAM9* mRNA expression levels, possibly as a result of the competition between the *ADAM5* pseudogene and the *ADAM9* gene for the miR-122b-5p binding site. *ADAM9* is known to be involved in tumor formation and progression, especially in HNSCC [42,43,44,45]. In addition, in salivary samples, lower expression of miR-122b-5p and higher expression of *ADAM9* were found to be a useful biomarker in the screening and diagnosis of oropharynx and oral SCC, respectively [44,46]. Moreover, miR-122b-5p has been found to interact with *DRAIC* lncRNA, transcribed from the 15q23 region, in nasopharyngeal SCC [47]. Our data suggest that gain of copies (more than three copies) of *ADAM3A* and *ADAM5* could lead to an increase of pseudogene-derived transcript that competes for miR-122b-5p, allowing for enhance of *ADAM9* expression and increasing OPSCC risk [42,43,44,45].

Furthermore, we found that OPSCC patients with total deletion or one copy of the *ADAM3A* and *ADAM5* pseudogenes presented lower EFS when compared with carriers of two or more copies. In the literature, total deletion of *ADAM3A* was associated with poor prognosis in early-stage non-small cell lung cancer patients [48]. We also found, using the Kaplan–Meier Plotter online database, that lower expression of the pseudogenes *ADAM3A* and *ADAM5* indicated worse RFS, and that higher expression of miR-122b-5p indicated lower OS in mixed HNSCC patients [35].

The contradictory results of *ADAM3A* and *ADAM5* CNV in OPSCC risk and prognosis could be explained by miR-122b-5p and therapy interaction. In agreement with our pseudogene-miRNA-mRNA hypothesis, the copy number loss of *ADAM5* leads to more available miR-122b-5p circulating, which could interact and silence other genes, including those involved in therapy modulation [21]. In lung cancer cells, miR-122-5p was found to be required for radiation-induced TGF-β activation and the epithelial-mesenchymal transition process, facilitating tumor cell invasion and migration [49]. It is worth noting that 95% of our patients cohort received irradiation treatment, thus, it is possible that miR-122-5p could be modulating patients’ therapy leading to a worse prognosis. In addition, high expression of miR-122b was identified in exosomes derived from HNSCC cells [50].

In summary, we identified for the first time that inherited abnormality CNV in the pseudogenes *ADAM3A* and *ADAM5* modulates the OPSCC occurrence and prognosis possible by *ADAM9* and miR-122b-5p interaction. We are aware of the study limitation, especially as the results were obtained in a relatively small number of patients from a single country and the lack of functional analysis to prove our pseudogene-miRNA-mRNA hypothesis. We highlight that this hypothesis must be validated with larger sample sizes and by functional analysis.

Nevertheless, our results can help researchers to identify new biomarkers for OPSCC diagnosis and to predict prognosis. Our study also contributes to target therapy development for patients’ treatment, especially for those that do not respond to conventional therapy.

## Figures and Tables

**Table 1:** Copy number variations in the *UGT2B17*, *ADAM3A* and *ADAM5*, and *SIRPB1* genes in 152 oropharyngeal squamous cell carcinoma patients and 155 controls.

CNV	OPSCC *n* (%)	Control *n* (%)	OR (CI 95%)	*p* Value
*UGT2B17* (4q13.2)				
0, 1 or 2 copies	152 (100.0)	150 (97.4)	NA	NA
3 or more copies	0 (0.0)	4 (2.6)		
0 or 1 copy	2 (1.3)	2 (1.3)	NA	1.00
2 or more copies	150 (98.7)	153 (98.7)		
*ADAM3A* and *ADAM5* (8p11.22)				
0, 1 or 2 copies	127 (83.6)	148 (95.5)	Reference	**0.002**
3 or more copies	25 (16.4)	7 (4.5)	6.49 (1.94–21.66)	
0 or 1 copy	49 (32.2)	47 (30.3)	Reference	0.29
2 or more copies	103 (67.8)	108 (69.7)	1.37 (0.75–2.50)	
*SIRPB1* (20p13)				
0, 1 or 2 copies	1 (0.7)	0 (0.0)	NA	NA
3 or more copies	151 (99.3)	155 (100.0)		
0 or 1 copy	0 (0.0)	1 (0.6)	NA	NA
2 or more copies	152 (100.0)	154 (99.4)		

CNV: copy number variation; OPSCC: oropharyngeal squamous cell carcinoma; *n*: number of cases; OR: odds ratio adjusted by age and alcohol consumption; CI: confidence interval; NA: not applicable. *p* value < 0.05 is presented in bold letters.

**Table 2:** Association of clinical–pathological characteristics and ADAM3A and ADAM5 copy number variations of 152 oropharyngeal squamous cell carcinoma patients.

Characteristic	*n*	*ADAM3A* and *ADAM5* CNV			
		0, 1 or 2 *n* (%)	3 or More *n* (%)	0 or 1 *n* (%)	2 or More *n* (%)
Tumor localization	152				
Base of tongue	72	59 (81.9)	13 (18.1)	21 (29.2)	51 (70.8)
Others	80	68 (85.0)	12 (15.0)	28 (35.0)	52 (65.0)
*p* value		0.61		0.44	
Histological grade	151 *				
Well or moderately	132	111 (84.1)	21 (15.9)	45 (34.1)	87 (65.9)
Poorly or undifferentiated	19	15 (78.9)	4 (21.1)	4 (21.0)	15 (79.0)
*p* value		0.52		0.30	
Nodal stage	152				
0	51	42 (82.4)	9 (17.6)	22 (43.1)	29 (56.9)
1 or more	101	85 (84.2)	16 (15.8)	27 (26.7)	74 (73.3)
*p* value		0.81		0.04 **	
Tumor stage	152				
I-III	49	43 (87.8)	6 (12.2)	20 (40.8)	29 (28.2)
IV	103	84 (81.6)	19 (18.4)	29 (59.2)	74 (71.8)
*p* value		0.48		0.11	

CNV: copy number variation; *n*: number of cases; others: tonsillar complex and soft palate. * The numbers of patients were not the same included in the study (*n* = 152), because no consistent information could be obtained from some individuals. ** *p* = 0.16 after false discovery rate adjustment.

**Table 3:** Clinical and pathological aspects and *ADAM3A* and *ADAM5* copy number variation in the event-free survival and overall survival of 139 oropharyngeal squamous cell carcinoma patients.

Variables	Event-Free Survival			Overall Survival		
	*n* Event/ *n* Total	*p* Value	HR (95% CI)	*n* Event/ *n* Total	*p* Value	HR (95% CI)
Age (years)						
≤57	48/78	0.40	1.21 (0.76–1.92)	58/78	0.27	1.24 (0.84–1.82)
>57	29/61		Reference	48/61		Reference
Gender						
Female	0/5	0.16	Reference	2/5	0.10	Reference
Male	77/134		22.08 (NA)	104/134		3.23 (0.79–13.11)
Histological grade						
Poor	11/19	0.54	1.22 (0.64–2.31)	14/19	0.81	1.07 (0.60–1.88)
Moderate	66/120		Reference	92/120		Reference
TNM stage						
I–III	18/44	0.003 *	Reference	29/44	**0.001**	Reference
IV	59/95		2.24 (1.31–3.82)	77/95		2.12 (1.37–3.26)
*ADAM3A* and *ADAM5*						
0, 1 or 2 copies	70/118	0.14	1.78 (0.82–3.88)	90/118	0.77	1.08 (0.63–1.84)
3 or more copies	7/21		Reference	16/21		Reference
0 or 1 copy	32/45	0.04 **	1.58 (1.01–2.48)	32/45	0.69	1.08 (0.71–1.64)
2 or more copies	45/94		Reference	74/94		Reference

*n*: number of patients; HR: hazard ratio; CI: confidence interval; NA: not applicable. Histological grade: Poor: poorly differentiated or undifferentiated, Moderate: moderately or well differentiated. Significant differences between groups are presented in bold letters. In multivariate Cox analysis (adjusted by TNM stage): * *p* = 0.002, HR: 2.34, 95% CI: 1.37–4.00; ** *p* = 0.02, HR: 1.68, 95% CI: 1.07–2.66.

## Supplementary Materials

The following supporting information can be downloaded at: https://www.mdpi.com/article/10.3390/genes13122408/s1, Table S1: Demographic and smoking and alcohol habits of 152 oropharyngeal squamous cell carcinoma patients and 155 controls; Table S2: Frequencies of tumor characteristics of 152 oropharyngeal squamous cell carcinoma patients; Table S3: Copy number variation sequences longer than 1 kb and identified at least in 5 patients associated with base of tongue squamous cell carcinoma risk in step 1 analyze; and Supplementary Dataset. Refs. [36,51,52,53,54,55,56] are cited in the Supplementary Materials.
