# Supplementary material for: Association of Inherited Copy Number Variation in ADAM3A and ADAM5 Pseudogenes with Oropharynx Cancer Risk and Outcome
Source: Genes (Basel). 2022 Dec 19;13(12):2408. doi: 10.3390/genes13122408 (PMC9778539; doi:10.3390/genes13122408)
Supplement: Supplementary file 1 [file genes-13-02408-s001.zip › Supplementary Information_revised.pdf]

Original Article

Association of inherited copy number variation in *ADAM3A* and *ADAM5* pseudogenes with oropharynx cancer risk and outcome

Juliana Carron[1], Caroline Torricelli[1], Janet Keller Silva[1], Yichuan Liu[2], Renata Pellegrino[3], Carmen Silvia Passos Lima[1,4], Gustavo Jacob Lourenço[1*]

[1]Laboratory of Cancer Genetics, School of Medical Sciences, University of Campinas, Campinas, São Paulo 13083-888, Brazil; julianacarron@outlook.com.br (J.C.); caroltorricelli@gmail.com (C.T.); janetkeller15@gmail.com (J.K.S.); carmenl@fcm.unicamp.br (C.S.P.L.)

[2]Center for Applied Genomics, The Children's Hospital of Philadelphia, Philadelphia, Pennsylvania PA 19104, USA; liuy5@email.chop.edu

[3]Department of Pediatrics, The Children's Hospital of Philadelphia, University of Pennsylvania, PA 19104, Philadelphia, Pennsylvania, PA 19104, USA; renata.pellegrino@gmail.com

[4]Department of Anesthesiology, Oncology, and Radiology, School of Medical Sciences, University of Campinas, Campinas, São Paulo 13083-887, Brazil

[*]Corresponding author

Gustavo Jacob Lourenço

Laboratory of Cancer Genetics, School of Medical Sciences, University of Campinas, Rua Vital Brasil, 50, Barão Geraldo, Campinas, São Paulo, postal code: 13083-888, Brazil. E-mail: guslour@unicamp.br

Table S1 Demographic and smoking and alcohol habits of 152 oropharyngeal squamous cell carcinoma patients and 155 controls

Characteristics	Number of patients (range or %)		Number of controls (range or %)
Median age (years)	56 (31-85)		46 (22-65)
p-value		**< 0.001**	
Gender			
Male	147 (96.7%)		149 (96.1%)
Female	5 (3.3%)		6 (3.9%)
p value		1.0	
Ethnic origin			
White	127 (83.6%)		128 (82.6%)
Nonwhite	25 (16.4%)		27 (17.4%)
p value		0.88	
Tobacco consumption			
Smokers	149 (98.0%)		41 (26.5%)
Non-smokers	3 (2.0%)		114 (73.5%)
p value		**< 0.001**	
Alcohol consumption			
Drinkers	141 (92.8%)		99 (63.9%)
Abstainers	11 (7.2%)		56 (36.1%)
p value		**< 0.001**	

Table S2 Frequencies of tumor characteristics of 152 oropharyngeal squamous cell carcinoma patients

Tumor characteristics	Number of patients (%)
Tumor size	
T1	9 (5.9)
T2	27 (17.8)
T3	43 (28.3)
T4	72 (47.4)
Not evaluated	1 (0.6)
Nodal stage	
N0	51 (33.6)
N1	30 (19.7)
N2	48 (31.6)
N3	23 (15.1)
Distant metastasis	
M0	145 (95.4)
M1	2 (1.3)
Not evaluated	5 (3.3)
Tumor stage	
I	7 (4.6)
II	10 (6.6)
III	32 (21.0)
IV	103 (67.8)
Histological grade	
Well-differentiated	13 (8.5)
Moderately-differentiated	119 (78.3)
Poorly-differentiated	17 (11.2)
Undifferentiated	2 (1.3)
Not evaluated	1 (0.7)
Tumor localization	
Base of tongue	72 (47.4)
Tonsillar complex	49 (32.2)
Soft palate	28 (18.4)
Uvula	2 (1.3)
Posterior pharyngeal wall	1 (0.7)

Table S3 Copy number variation sequences longer than 1 kb and identified at least in 5 patients associated with base of tongue squamous cell carcinoma risk in step 1 analyze

Chromosome	Position	Gene	Function
4	69,085,497-69,116,840	*UGT2B17*	Detoxification (Bhoi et al. 2016)
8	39,291,338-39,517,385	*ADAM3A* and *ADAM5*	Pseudogenes. *ADAM* family function: cell-to-cell communication and adhesion (Barrow et al. 2011)
11	55,134,451-55,209,190	*OR4S2*	Olfactory receptor (Diels et al. 2020)
15	18,866,633-19,788,509	*POTEB*	Protein-protein interaction (Lee et al. 2006)
15	32,496,931-32,576,764	*GOLGA8A*	Protein and lipid transport (Wang et al. 2018)
17	41,513,416-41,757,121	*KANSL1-AS1*	Long non-coding RNA. Unknown
20	1,525,985-1,548,689	*SIRPB1*	Cell proliferation (Song et al. 2020)
22	22,683,317-22,716,131	*GSTT1*	Detoxification (Masood et al. 2014)

References

Bhoi S, Baliakas P, Cortese D et al (2016) UGT2B17 expression: a novel prognostic marker within IGHV-mutated chronic lymphocytic leukemia? Haematologica 101(2):e63-65. https://doi.org/10.3324/haematol.2015.136440

Barrow J, Adamowicz-Brice M, Cartmill M et al (2011) Homozygous loss of ADAM3A revealed by genome-wide analysis of pediatric high-grade glioma and diffuse intrinsic pontine gliomas. Neuro Oncol 13(2):212-222. https://doi.org/10.1093/neuonc/noq158

Diels S, Huybreghts S, Van Hoorenbeeck K et al (2020) Copy number variant analysis and expression profiling of the olfactory receptor-rich 11q11 region in obesity predisposition. Mol Genet Metab Rep 25:100656. https://doi.org/10.1016/j.ymgmr.2020.100656

Lee Y, Ise T, Ha D et al (2006) Evolution and expression of chimeric POTE-actin genes in the human genome. Proc Natl Acad Sci USA 103(47):17885-17890. https://doi.org/10.1073/pnas.0608344103

Wang Y, Cheng C, Zhang Z et al (2018) Blood-based dynamic genomic signature for obsessive-compulsive disorder. Am J Med Genet B Neuropsychiatr Genet 177(8):709-716. https://doi.org/10.1002/ajmg.b.32675

Song Q, Qin S, Pascal LE et al (2020) SIRPB1 promotes prostate cancer cell proliferation via Akt activation. Prostate 80(4):352-364. https://doi.org/10.1002/pros.23950

Masood N, Yasmin A, Kayani MA (2014) Genetic variations and head and neck cancer risks. Mol Biol Rep 41(4):2667-2670. https://doi.org/10.1007/s11033-014-3125-6
